# Experimental Study on Phenol-Formaldehyde Resin Aggregates as In-Depth Conformance Control Agents Stabilized by Polymer

**DOI:** 10.3390/polym14153159

**Published:** 2022-08-03

**Authors:** Xianxing Meng, Guiqing Zhang, Jian Wu, Xiong Zhao, Lin Wang, Fang Zhang

**Affiliations:** 1 School of Chemistry and Chemical Engineering, Ankang University, Ankang 725000, China; huobin1984@126.com; 2 Ankang Research Centre of New Nano-Materials Science and Technology, Ankang University, Ankang 725000, China; 3 CNPC Engineering Technology Research Co., Ltd., Tianjin 300451, China; zhanggq2@cnpc.com.cn; 4 Changqing Downhole Technology CoMPany, CNPC Chuanqing Drilling Engineering Co., Ltd., Xi’an 710000, China; cj_wujian@cnpc.com.cn; 5 Shixi Production Plant, Xinjiang Oilfield Company, PetroChina, Karamay 834000, China; sxytzhaox@petrochina.com.cn; 6 Karamay Drilling Company of CNPC Xibu Drilling Engineering Company Co., Ltd., Karamay 834000, China; kzwanglin@cnpc.com.cn

**Keywords:** phenol-formaldehyde resin, molecular aggregates, dispersion stability, in-depth conformance control, improved oil recovery

## Abstract

To improve the dispersion stability of phenol-formaldehyde resin (PFR) particles in simulated oilfield injection water and their propagation ability in petroleum reservoir, a hydrophobically associating polymer (HAP) was employed as a stabilizer in this paper. The dispersion stability of PFR in the injection water was studied by measuring turbidity as a function of time. In addition, the migration property of the PFR/HAP dispersion was evaluated by both cellulose membrane filtration and sand packs-flooding experiments. The results show that HAP can stabilize the PFR dispersion prepared with the simulated injection water by forming PFR/HAP complex molecular aggregates. These aggregates can migrate in sand packs with strong flow resistance due to deformation or disaggregation of the aggregates when passing through the pore throat. Oil recovery was improved by up to 21.1% on the basis of water flooding, and the higher the concentration of PFR/HAP dispersion system, the better the oil recovery effect. Moreover, the cycle of log-jamming/dispersion of the aggregates leads to their penetrations through the bigger pores in the sand packs with a higher flow resistance than water. This process can improve the conformance of water in high permeability sand packs on a micro/macro scale and thus divert more water into low permeability sand packs. Therefore, more oil could be recovered from the low permeability sand packs. Moreover, the bigger the sand pack’s permeability ratio, the lower the oil recovery rate by waterflood, and the more the incremental oil can be recovered by the PFR/HAP flood.

## 1. Introduction

Petroleum resources are still the main source of global energy and play an important role in all of the aspects of people’s daily lives and social development. The average oil recovery rate is between 30% and 60% with current development technology [1,2]. Water injection is an effective method to produce oil, after the primary recovery which uses reservoir pressure to extract the oil [3]. However, waterflooding typically yields only 30 to 40 percent of the oil, and only a fraction of the geological reserves can be recovered, with most of the oil remaining in the original reservoir. The injection of chemicals, including polymers [4,5], alkalis [6], and surfactants [7], enhances recovery by improving the water–oil fluidity ratio, altering wettability, or reducing the oil–water interfacial tension. Polymer flooding is the most widely used of these technologies. Polymers [8,9] reduce the oil–water flow ratio and increase the sweep volume, thereby improving the viscosity recovery. At present, polymers have been widely used in many oil fields and achieved some results. However, polymers have a poor shear resistance [10] and temperature resistance [11], and their viscosity-increasing effect is limited, which limits their ability to increase the sweep volume. In addition, the dominant channels of water flow will be formed after the water flooding [12], which will change the pore structure of the reservoir. All of these factors result in limited enhanced oil-recovery capabilities of the polymer flooding. As a result, polymer flooding can only improve recovery by about 10% over water flooding [13].

In recent years, nanomaterials have attracted people’s attention because of their unique physical and chemical properties [14,15]. Nanomaterials, typically defined as suspensions with solid particles smaller than 100 nm, have the advantages of a high heat transfer capacity, large surface area, and low erosion and pressure drop compared to conventional suspensions, especially in microchannels. Many researchers have studied the application of nanomaterials alone or in combination with surfactants and polymers to enhance oil recovery [16,17], to delay the abandonment of old oil fields.

After extensive research and testing, the nanomaterials based on the particles formed by PFR molecular aggregates were proposed for enhanced oil recovery (EOR) [18,19]. PFR is a water-soluble phenolic resin with a polymerization degree of 2~10 and a relative molecular weight of 200~1200. The benzene rings in the molecule are linked by methylene via para–para and ortho–para positions [20]. When the PFR molecules were dispersed in water, they spontaneously aggregated in a solution due to intermolecular forces. However, the PFR dispersion stability is easily affected by the ionic strength, especially the high-valence ions, such as calcium ions or magnesium ions. At high ionic strength, the PFR molecules rapidly aggregate into larger particles, and its deep transport capacity in porous media decreases significantly.

Hydrolyzed polyacrylamide (HPAM) is the most commonly used polymer in enhanced oil recovery (EOR) applications. In EOR, the polymers are added to the displacement water to increase its flow resistance, resulting in a favorable mobility ratio [21]. HAP is currently attracting increasing attention from polymer EOR researchers because of its ability to produce a higher flow resistance than HPAM at lower concentrations, better salinity and temperature resistance [22]. HAP contains a number of more or less randomly distributed hydrophobic groups. The individual hydrophobic segments in HAP tend to self-associate intramolecularly or intermolecularly, strongly affecting the viscosity of the aqueous solutions, making HAPs useful viscosity modifiers. Moreover, the introduction of hydrophobic groups into other water-soluble substances also produces local amphiphilism in the structure, and the resulting surface activity of the amphiphilic fragments can be regulated over a wide range. This has led to HAPs being widely used as a reagent to remove unwanted particles or chemicals from contaminated water, or to dissolve substances with a low solubility into solutions or colloidal suspensions.

In this work, the HAP was used to increase PFR dispersion in simulated injection water. The effect of the HAP on the dispersion stability of the PFR molecules in water was investigated by measuring the turbidity of the dispersion system. The microporous membrane filtration experiments were conducted to evaluate the plugging characteristics of the PFR/HAP dispersion. The transport characteristics in the porous media of the PFR/HAP dispersion were studied in the packed sand-pack which has three pressure sensors. Parallel sand-tube flooding experiments were carried out, to inspect the oil displacement effect of the PFR/HAP dispersion.

## 2. Materials and Methods

### 2.1. Materials

Phenol-formaldehyde resin (20 wt.%) was synthesized in our lab, and the chemical properties of the PFR were reported in our previous work [20]. Hydrophobically associating polymer (HAP) was supplied by the Daqing oilfield. The HAP solution with a concentration of 0.2 wt.% was prepared by dissolving the dry powder of HAP in deionized water, aging at room temperature for 48 h before use. Simulated injection water has a salinity of 5196 mg/kg; the ion composition of the simulated injection water is shown in Table 1. All of the chemicals used in this study are analytical grade. The simulated oil, which has a viscosity of 10.5 mPa·s at 45 °C, was prepared by mixing jet fuel and crude oil supplied by the Daqing oil field. The density of the simulated oil was 0.8554 g/cm^3^ at 45 °C. The cellulose membrane was purchased from China Nantong Longjin Membrane Technology Co., LTD., with a diameter of 47 mm and an average aperture of 5 μm. 

### 2.2. Methodology

#### 2.2.1. Measurement of PFR Solution Turbidity

The turbidity of PFR solutions was measured by a turbidity meter (Turb 550, WTW, Munich, Germany) at 25 °C. The measurement principle of turbidity is based on light scattering. A tungsten lamp was used as a light source with a measurement range from 0.01 to 1000 NTU.

#### 2.2.2. Cellulose Membrane Filtration

**Figure 1:**
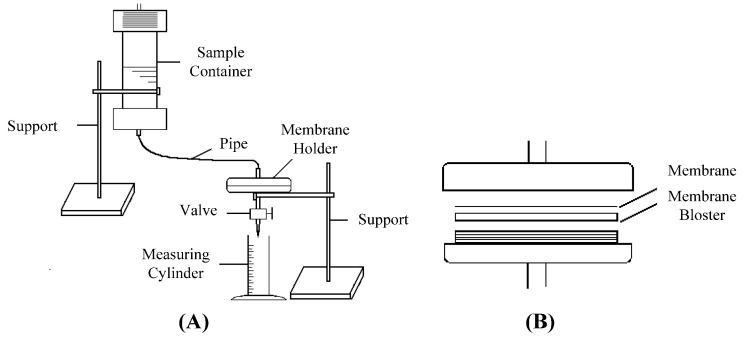
Schematic diagram of filtration device: (**A**) set for membrane blocking; (**B**) membrane holder.

**Figure 2:**
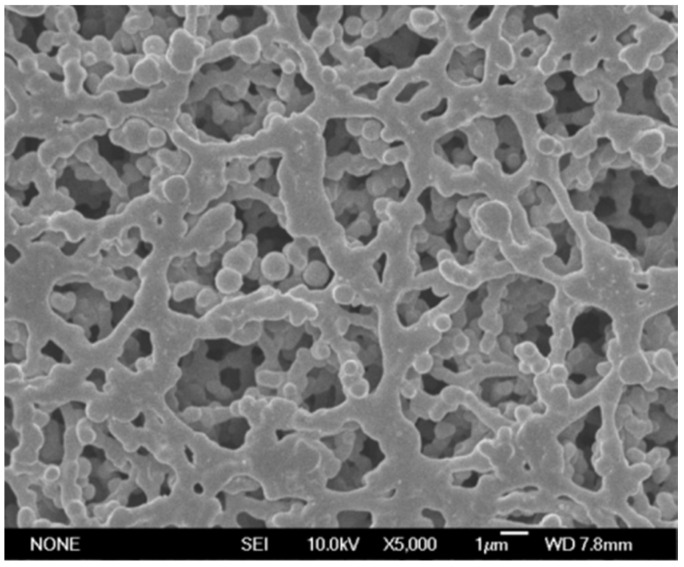
SEM photo of cellulose membrane.

A schematic diagram of the membrane filtration device is shown in Figure 1. The filtration time and filtration volume were recorded during the filtering of 20 mL of PFR solution or PFR/HAP dispersion through a membrane under the pressure of 0.1 MPa, which was provided by an air compressor. Figure 2 gives a scanning electron micrograph of the membrane.

#### 2.2.3. PFR/HAP Migration Behavior in the Sand Pack

**Figure 3:**
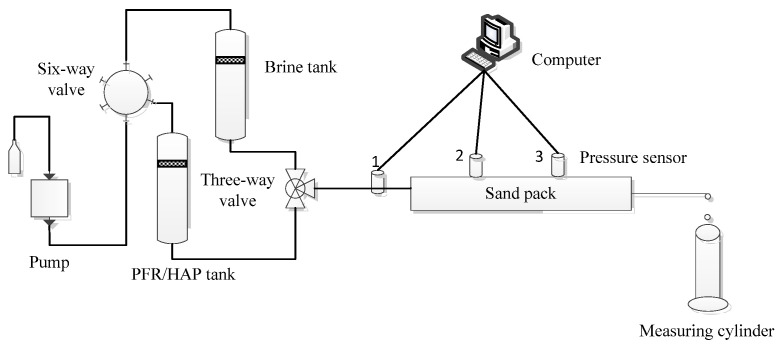
Schematic diagram of flood experiment for a sand pack.

A sand pack of 50 cm in length and 2.5 cm in the inner diameter of a steel tube was used in this study, which has three pressure sensors monitoring the pressure gradient along with the sand pack. The first sensor was located at the entrance of the sand pack and the other two were installed on the sand pack, which equally divided the sand pack into three parts. Two of the sand pack experiments were performed to evaluate the blocking and penetration properties of the PFR/HAP dispersion in porous media at room temperature. Figure 3 shows the schematic diagram of the sand pack flood. The sand pack preparation was as follows: a small number of quart particles were added into the steel tube; followed by adding a small amount of the simulated injection water; then the sand pack was vigorously beaten with a wooden stick along the peripheral steel tube; the quartz particles were compacted with a steel rod into a diameter of less than the inner diameter of the steel tube; the above steps were repeated until the steel tube was fully packed with quartz particles. The pore volume was calculated as the difference between the inner volume of the steel tube and the volume of the filled quartz particles. The volume of the quartz particles filled in the steel tube was calculated by the mass of the particles divided by the quartz particles’ density, which was 2.675 g/cm^3^. The permeability of the sand pack was measured with the injection of the simulated injection water at 0.4 mL/min. The permeability of the sand pack can be calculated, according to Darcy’s law:

(1) $$K=\frac{Q\mu L}{A\Delta P}$$

where, *A* is the sand pack cross-section area (cm^2^); *Q* is the fluid injection flow rate(cm^3^/s); *μ* is fluid viscosity (mPa·s); Δ*P* is the differential pressure at both ends of the sand pack; and *L* is sand pack length (cm).

For each experiment, the sand pack was injected with the simulated injection water at 0.4 mL/min until the injection pressure was stable. Then, the sand pack was injected with the PFR/HAP dispersion at 0.4 mL/min for about 0.5 pore volume (PV). Finally, the simulated injection water was injected at the same flow rate for at least 2 PV. The injection pressure at each pressure sensor along the sand pack was monitored by a computer throughout the whole experiment.

#### 2.2.4. Oil Displacement in Parallel Sand Packs

**Figure 4:**
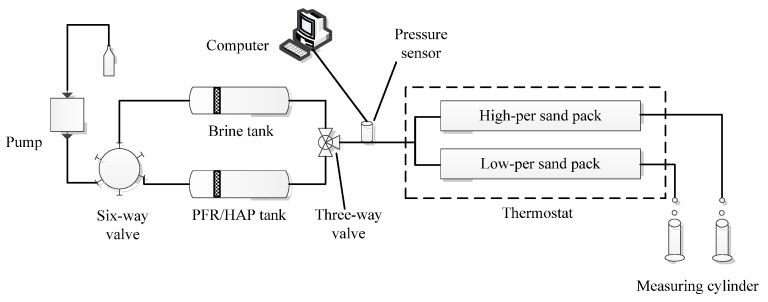
Schematic diagram of oil displacement experiment for parallel sand packs.

Five parallel sand packs were conducted in this study, to evaluate the oil displacement performance of the PFR/HAP dispersion in the heterogeneous reservoirs. The schematic diagram of the parallel sand packs is shown in Figure 4. The sand packs had an inner diameter of 2.5 cm and a length of 30 cm of steel tubes. The two sand packs with different permeabilities were prepared separately before the flooding process. First, the sand pack was dry packed with the same procedure as the preparation of the sand pack described in Section 2.2, except the simulated injection water was not added. The sand pack was vacuumed for 4 h and then saturated with the simulated injection water. The pore volume of the sand pack was determined as the volume of the water saturated. Then the sand pack was injected with the simulated injection water at 0.4 mL/min until the injection pressure was stable and the water permeability was calculated. After the measurement of the permeability, the sand pack was flooded with simulated oil at 0.2 mL/min, until no more water came out of the sand pack. The saturated oil volume in the sand pack was determined as the volume of the water obtained from the sand pack.

After being saturated with the oil, two sand packs with different permeabilities were installed, according to the setup shown in Figure 4. The pressure sensor was located at the inlet of the two parallel sand packs. First, the simulated water was injected at 0.4 mL/min until the water cut up to 98%, followed by flooding with the PFR/HAP dispersion at 0.4 mL/min for 0.5 PV. Then, the parallel sand packs were injected with the simulated injection water at the same flow rate. The water injection was stopped when no more oil came out of the sand packs. During the whole flooding experiment, the produced fluid from the two sand packs was collected separately, by measuring the cylinder at the same time intervals. The water cut and oil recovery rate were determined by measuring the produced fluid.

## 3. Results

### 3.1. PFR Dispersion Stability

**Figure 5:**
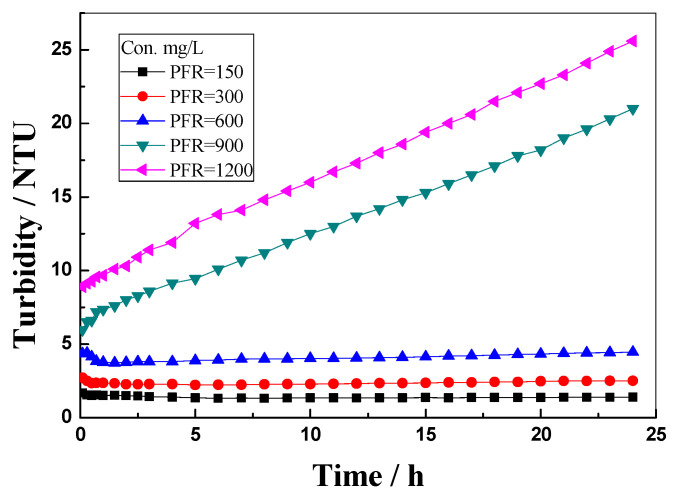
Effect of concentration on the turbidity of PFR solution prepared with simulated injection water at 25 °C.

Dispersion stability is an important factor for evaluating the profile control agents because it can influence the blocking and penetration performance when migrating in porous media. The turbidity evaluation over time was conducted to figure out the dispersion stability of the PFR solution in the simulated injection water with the PFR concentration in the range of 150~1200 mg/L, as shown in Figure 5. It is observed that, when the PFR concentration was in the range of 150 to 600 mg/L, the turbidity remained unchanged for 24 h, indicating that the PFR dispersion was stable. When the PFR concentration was higher than 600 mg/L, however, the turbidity of the PFR dispersion prepared with the simulated injection water increased with time. This result shows that the PFR dispersion became unstable as more or larger molecular aggregates were formed in the simulated injection water.

**Figure 6:**
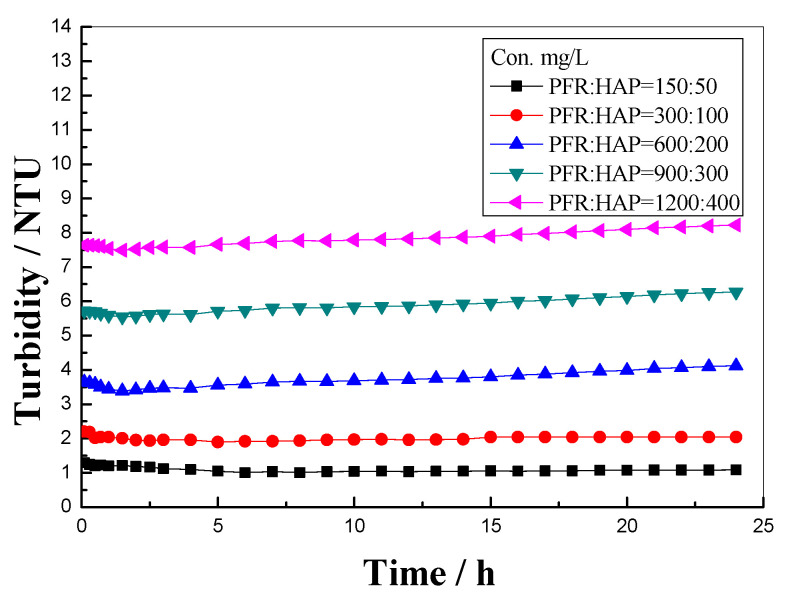
Effect of HAP on the turbidity of PFR prepared with simulated injection water at 25 °C.

To study the effect of the HPA on the PFR dispersion stability, the turbidity of the PFR/HAP mixture solution prepared with simulated injection water versus time was evaluated, as shown in Figure 6. The molar ratio of the PFR:HAP was 3:1 for all of the PFR/HAP mixture solutions. It can be seen that the turbidity of the PFR/HAP mixture solutions with the PFR concentration of 150~300 mg/L remained unchanged for 24 h. When the PFR concentration was higher than 300 mg/L, the turbidity of the PFR/HAP mixture solutions slightly increased with time, and the turbidity was all below 9 NTU in the whole experiment process. These results show that the PFR/HAP mixture solution was stable in the simulated injection water and proves that HAP can stabilize the PFR dispersion in the simulated injection water. The stabilizing ability of HAP on the PFR aggregates contributed to the formation of the complex molecular aggregates of PFR/HAP molecules, because of a similar molecular structure of the PFR and HAP molecules. The complex molecular aggregates have more charge density on the surface than that of the PFR aggregates, which resulted in a higher repulsive force between the complex molecular aggregates, hence the PFR/HAP mixture solutions were more stable than the PFR solution in simulated injection water.

### 3.2. Blocking Property of PFR/HAP and HAP Solution in Cellulose Membrane

**Figure 7:**
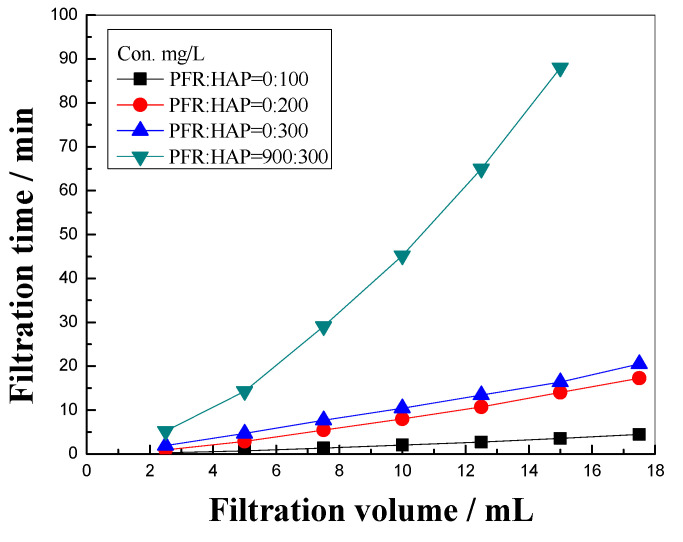
Filtration volume vs. filtration time curve of PFR/HAP dispersion and HAP solution prepared with simulated injection water.

The membrane filtration experiment is an effective and convenient method for evaluating the blocking property of the chemical agents in porous media [23]. Figure 7 shows that the filtration time for the HAP solution with a concentration in the range of 100~300 mg/L increased linearly with the filtration volume, which indicates that the HAP solution cannot block the cellulose membrane with an average pore diameter of 5 μm. Due to the HPA molecular chains being more flexible, the polymer solution can easily flow through the membrane without blocking the cellulose membrane, even though the viscosity of the solution was increased with the increasing concentration. When the PFR (900 mg/L) was added to the HAP solution, the PFR/HAP mixture solution took a much longer time than the HAP solution which had the same HAP concentration, when the filtering volume of the solutions was the same. This result demonstrates that the PFR/HAP mixture solution can block the membrane effectively, due to the formation of complex molecular aggregates of PFR and HAP. 

### 3.3. Flow Behavior in Sand Packs

**Figure 8:**
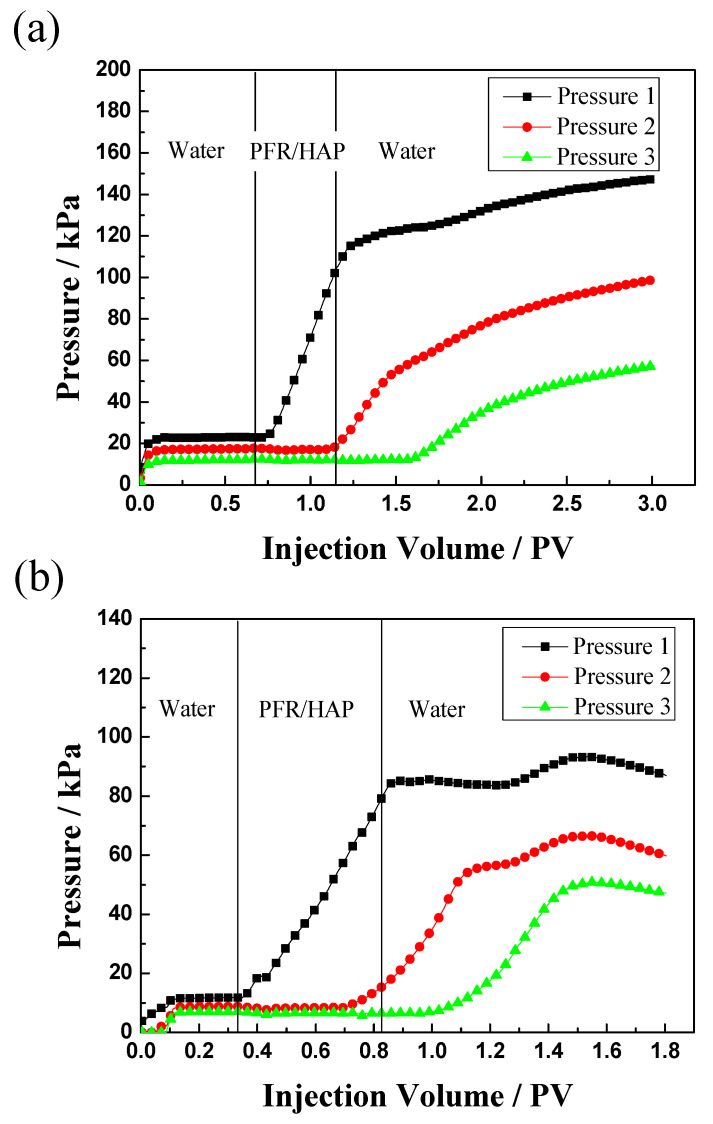
Injection pressure as a function of injection volume for PFR/HAP dispersion flood for sand packs ((**a**): K: 380 mD, PFR:HAP 300:100 mg/L; (**b**): K:1000 mD, PFR:HAP 1200:400 mg/L).

Two sand pack experiments were conducted to evaluate the flow behavior of the PFR/HAP dispersion prepared by simulated injection water in porous media. The sand pack permeability was 380 mD and 1000 mD flooded with the PFR/HAP dispersion at a concentration of 300/100 mg/L and 1200/400 mg/L, respectively. Figure 8 shows clearly that the pressure of injecting the PFR/HAP dispersion and chase water was much higher than the initial water injection and the pressure at three measuring points along with the sand packs changed in a similar trend with pressure 1 > pressure 2 > pressure 3. These phenomena demonstrate that the PFR/HAP complex aggregates can migrate with high resistance in the sand packs, due to the deformation or disaggregation of the aggregates when passing through the pore throat. The reason that this happened is probably that the pressure difference crossing the pore throat was less than the resistance of the aggregates on the pore throat; the PFR/HAP molecular aggregates accumulated and partially blocked the pore throat, which resulted in the log-jamming of the aggregates. When the pressure difference crossing the pore throat was increasing and was higher than the resistance of the aggregates on the pore throat, the PFR/HAP molecular aggregates were deformed or disaggregated and penetrated through the pore throat, and then dispersed into the next pore throat. The cycle of log-jamming/dispersion of the aggregates leads to the aggregates penetrating through the bigger pores in the sand pack with a much higher flow resistance than water. Compared with Figure 8a,b, it is clear that the sand pack with a higher permeability also needs a higher concentration of the chemicals to build up the pressure.

### 3.4. Oil Displacement in Parallel Sand Packs

To study the oil displacement performance of the PFR/HAP dispersion prepared with the simulated injection water, five parallel sand packs with different permeability ratios (high-perm to low-perm ratio was 2.1~11.3, the permeability of the low-perm sand packs was 180~250 mD) were conducted at 45 °C. Table 2 shows that the oil recovery rate of the initial water flooding was 30.7~58.7% and decreased as the permeability ratio increased. This is mainly due to the fraction of the injected water flowing through the low-perm sand pack being decreased as the permeability ratio increased. With the PFR/HAP dispersion flood, the incremental oil recovery rate was increased from 10.9% to 21.1% as of the permeability ratio increased. It should be noted that the incremental oil was mainly from the low-perm sand pack, with the incremental oil recovery rate increasing from 13.8% to 31.3% as the permeability ratio increased for test1 to test4. It is obvious that as the log-jamming/dispersion of the PFR/HAP molecular aggregates in the high-perm sand packs increased the flow resistance, without blocking the low-perm sand packs, and diverted more water into the low-perm sand packs, this led to more oil flooding out from the low-perm sand packs. Table 2 also shows that the incremental oil is not only from the low-perm sand packs but also from the high-perm sand pack, with an incremental oil recovery rate of 6.2–11.1%. This result indicates that the log-jamming/dispersion of the PFR/HAP molecular aggregates in the high-perm sand packs can also improve the flow profile of the water in the high-perm sand packs on the micro/macro scale. 

**Figure 9:**
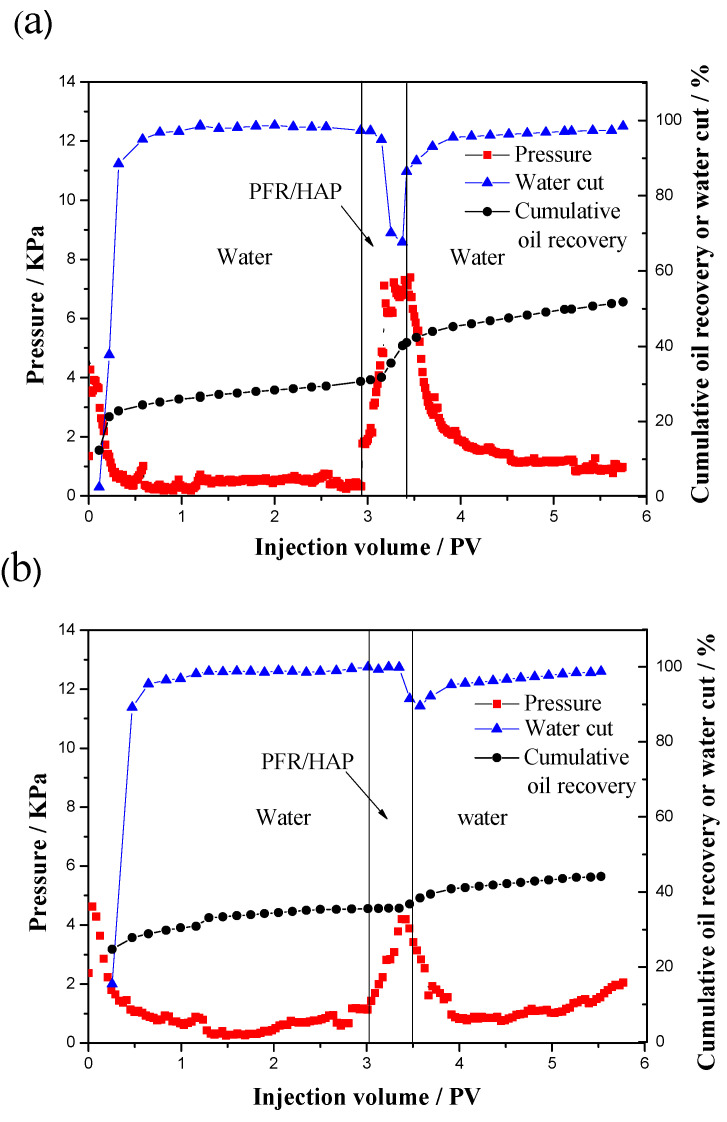
Pressure, water cut and cumulative oil recovery as a function injected volume for PFR/HAP dispersion flood of parallel sand packs ((**a**): test4; (**b**): test5).

Comparing test4 with test5, both of the tests have almost the same permeability ratio of the sand packs and permeability of the low-perm sand packs. However, test4 had a higher incremental oil recovery rate of 21.1% with a higher concentration of PFR/HAP solution (1800:600 mg/L) flooding. The polymer flooding has been used in the Daqing oilfield in northeast China since 1996, and the enhanced oil recovery rate is about 13~14% [24]. At a high concentration, the PFR/HAP solution flooding improved the recovery by more than 20%, significantly higher than the polymer flooding. Figure 9 shows the pressure, water cut, and cumulative oil recovery as a function of the injected fluid volume of test4 and test5. The pressure during the injection of the PFR/HAP solution increased more and the water cut decreased more obviously with a higher PFR/HAP concentration for test4. These results show that the higher the PFR/HAP concentration, the higher the pressure, the more the incremental oil recovered and the bigger the decrease in the water cut for the same sand packs’ permeability ratio. The results of test4 and test5 also show that, as the permeability ratio is up to 11.1~11.3, the oil in the low-perm sand packs was almost not flooded out by water and the oil recovery rate was 30.7% and 35.5%, which is relatively low compared with test1 to test3. These results prove that the higher the reservoir heterogeneity, the lower the oil recovery rate is for the waterflood. 

## 4. Discussion

In the deep profile control system, the deep migration capacity is an important guarantee for improving oil recovery. The attraction between the PFR molecules in an aqueous solution includes the hydrogen bond, π–π interaction and the van der Waals interaction, and the repulsive interaction includes electrostatic repulsion [20]. The mutual effect of the repulsive force and the attractive force determines the size of its aggregation in the aqueous solution [25]. Attractions (hydrogen bonding, π–π interactions and van der Waals interactions) are less affected by ionic strength. On the other hand, the compression of the electrostatic diffusion double layer by the anti-sign ions makes the electrostatic repulsion significantly decrease at a high ionic strength. Therefore, at a high ionic strength, the formed molecular aggregates will be larger and even lose the dispersion stability. Furthermore, the large PFR molecular aggregates are not easy to migrate deep into the porous medium [19]. The presence of the HAP improves the dispersion stability of the PFR aggregates in the salt solution. This is mainly because the HAP can combine with the phenolic resin molecules through hydrogen bonding, van der Waals interaction and hydrophobic association. The kinetic energy of the PFR molecular aggregates attached to the polymer chain is reduced, which is not conducive to the combination of the molecular aggregates overcoming the repulsive energy barrier through collisions. At the same time, the existence of the polymer chain also protects the molecular aggregates physically and increases the stability of the molecular aggregates. Based on the above reasons, the phenolic resin molecular aggregates not only have a good dispersion stability, but also their deep migration ability in porous media is significantly improved.

## 5. Conclusions

The HAP can stabilize the PFR prepared with the simulated injection water by the formation of PFR/HAP complex molecular aggregates. The PFR/HAP mixed dispersion system can effectively block the microporous membrane, but the polymer solution with the same polymer concentration cannot block the membrane. The aggregates can migrate into sand packs with high resistance due to the deformation or disaggregation of the aggregates when passing through the pore throat. The cycle of the log-jamming/dispersion of the aggregates leads to the aggregates penetrating through the bigger pores in the sand packs with a higher flow resistance than water. This process can improve the flow profile of water in the high-perm sand packs on a micro/macro scale and divert more water into low-perm sand packs, therefore, there was more oil that flooded out from the low-perm sand packs. When the permeability ratio was 11.3, the oil displacement increased up to 21.1% in the parallel sand packs with a high concentration (PFR:HAP 1800:600 mg/L). The higher the sand packs’ permeability ratio, the lower the oil recovery rate by waterflood, and the more the incremental oil that was recovered with the PFR/HAP flood.

## Figures and Tables

**Table 1:** Composition of simulated injection water.

Ion	Na^+^	Ca^2+^	Mg^2+^	Cl^−^	CO_3_^2−^	HCO^3−^	SO_4_^2−^	Total
Concentration/mg/L	1686.8	0.7	0.8	1214.3	96.8	2196.0	0.7	5196.3

**Table 2:** Oil recovery of PFR/HAP dispersion flood for parallel sand packs.

Test	Sand Pack	K/mD	Perm. Ratio	PFR: HAP/ mg/L	RFw/%		Incremental Oil Recovery/%	
					Single	Total	Single	Total
1	High-perm	480	2.1	510:170	64.3	58.7	8.4	10.9
	Low-perm	230			52.6		13.8	
2	High-perm	1250	5.0	600:200	63.6	52.1	6.2	18.3
	Low-perm	250			41.0		30.0	
3	High-perm	1630	8.1	1200:400	63.1	48.2	7.3	19.2
	Low-perm	200			34.3		30.2	
4	High-perm	2220	11.1	1800:600	58.2	30.7	11.1	21.1
	Low-perm	200			1.7		31.3	
5	High-perm	2030	11.3	900:300	63.4	35.5	8.7	8.4
	Low-perm	180			0.0		8.0	

## Data Availability

Not applicable.
